# Quartz Tuning Fork Sensor-Based Dosimetry for Sensitive Detection of Gamma Radiation

**DOI:** 10.3390/ma14227035

**Published:** 2021-11-19

**Authors:** Nadyah Alanazi, Abdullah N. Alodhayb, Atheer Almutairi, Hanan Alshehri, Sarah AlYemni, Ghadah Alsowygh, Sabaa Abdulmawla, Khaled Shamma, Hamad Albrithen, Muthumareeswaran Muthuramamoorthy, Aljawhara H. Almuqrin

**Affiliations:** 1 Department of Physics and Astronomy, College of Science, King Saud University, Riyadh 11451, Saudi Arabia; nalenazi@ksu.edu.sa (N.A.); aalodhayb@ksu.edu.sa (A.N.A.); 438202676@student.ksu.edu.sa (A.A.); hoalshehri@KSU.EDU.SA (H.A.); salyemni@KSU.EDU.SA (S.A.); galsowygh@ksu.edu.sa (G.A.); stabdulmawla@ksu.edu.sa (S.A.); khaledshamma@hotmail.com (K.S.); brithen@ksu.edu.sa (H.A.); 2 Research Chair for Tribology, Surface, and Interface Sciences, Department of Physics and Astronomy, College of Science, King Saud University, Riyadh 11451, Saudi Arabia; 3 King Abdullah Institute for Nanotechnology, King Saud University, Riyadh 11451, Saudi Arabia; mramamoorthy@ksu.edu.sa; 4 K.A.CARE Energy Research and Innovation Center at Riyadh, Riyadh 11451, Saudi Arabia; 5 Department of Physics, College of Science, Princess Nourah bint Abdulrahman University, Riyadh 11564, Saudi Arabia

**Keywords:** dosimetry, gamma rays, quartz tuning fork, radiation sensing

## Abstract

This study generally relates to nuclear sensors and specifically to detecting nuclear and electromagnetic radiation using an ultrasensitive quartz tuning fork (QTF) sensor. We aim to detect low doses of gamma radiation with fast response time using QTF. Three different types of QTFs (uncoated and gold coated) were used in this study in order to investigate their sensitivity to gamma radiations. Our results show that a thick gold coating on QTF can enhance the quality factor and increase the resonance frequency from 32.7 to 32.9 kHz as compared to uncoated QTF. The results also show that increasing the surface area of the gold coating on the QTF can significantly enhance the sensitivity of the QTF to radiation. We investigated the properties of gold-coated and uncoated QTFs before and after irradiation by scanning electron microscopy. We further investigated the optical properties of SiO_2_ wafers (quartz) by spectroscopic ellipsometry (SE). The SE studies revealed that even a small change in the microstructure of the material caused by gamma radiation would have an impact on mechanical properties of QTF, resulting in a shift in resonance frequency. Overall, the results of the experiments demonstrated the feasibility of using QTF sensors as an easy to use, low-cost, and sensitive radiation detector.

## 1. Introduction

The increasing use of radioactive sources in several applications, including medicine, research, education, industry, and agriculture, has significantly increased the demand for inexpensive, accurate, and portable devices for detecting nuclear radiation [1,2]. Alternative detection techniques are needed due to the health dangers associated with high-energy ionizing electromagnetic radiation, such as gamma rays. Gamma rays, unlike alpha and beta particles, can easily permeate the body, posing health dangers such as tissue and organ damage [3]. Minimizing radiation exposure is one of the most basic strategies to safeguard one’s health from radiation [4]. Therefore, in this approach, we aim to detect low doses of gamma radiation with fast response time using a quartz tuning fork (QTF). Although there are other devices for detecting nuclear radiation with high accuracy, such as gas-filled, scintillation, and semiconductor detectors [5,6,7], the proposed approach is a good alternative capable of providing ultrasensitive, rapid, cost-efficient, and small-size detection of nuclear radiation.

QTF-based sensors have recently gained significant interest owing to their attractive characteristics, such as high mechanical stability, quality factor, fast response time, and low cost, compared to conventional devices, including surface acoustic wave, quartz crystal microbalance, and microcantilever sensors [8]. These inherent properties of QTF have been harnessed in many applications, such as in detecting several physical and chemical properties, including density, viscosity, pressure, gas sensing, and temperature [9,10,11,12,13].

The detection of gamma rays using different sensing devices and techniques, including polymer-based fiber Bragg grating sensors [14], MOS capacitors [15], and fluorescence quenching [16], has been reported. Shimoda et al. [17] investigated the relationship between frequency shift and gamma radiation. They reported that quartz resonators experienced a frequency shift when irradiated with high doses of gamma rays (up to 7.5 × 10^6^ rad). This is attributed to the defects in quartz. The study provides a basis for quartz-based sensors for measuring radiation. Accordingly, there is a need for more sensitive and low-cost techniques for detecting nuclear and electromagnetic radiation. In this study, we employ QTFs, a low-cost class of crystal oscillators capable of providing orthogonal measurements, to investigate their potential in detecting radiations. Although micro-electrical mechanical systems (MEMS), such as microcantilever sensors, have been used to detect radiation energy with high sensitivity, the high cost of sensing chips remains an issue [18,19,20]. Hence, QTF is an alternative device for sensing radiation energy owing to its high quality factor (Q), cost-effectiveness, and fast response time with a simple operating circuit system [21]. The importance of this study stems from the fact that gaining a deeper insight into the response of QTFs with its appealing characteristics to gamma radiation is key for developing other crystal-based sensing techniques for radiation detection. It also investigates their potential for continuous monitoring of radiation in small rooms or laboratories where radiation, especially low-dose radiation, may be present.

## 2. Experimental Details

**Figure 1:**
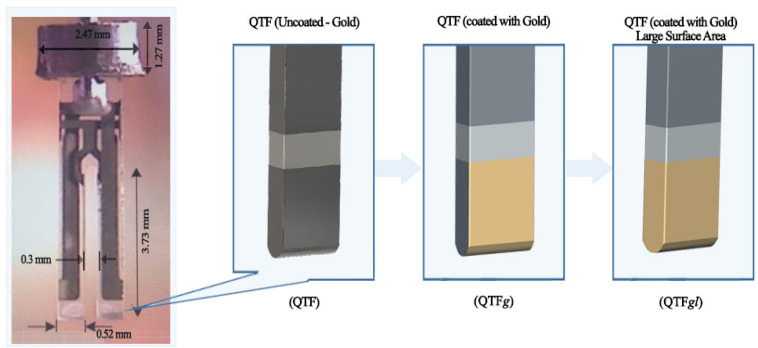
Schematic representation of QTF, QTF*g*, and QFT*gl*.

The QTF had two vibrating prongs made of quartz substrate connected at one end, costing ~1 USD/piece. Each prong was 3.73 mm long and 0.52 mm wide, as shown in Figure 1. The QTF used in this work was composed of quartz with a thickness of 300 nm with silver electrodes placed on the sides of the tuning prongs [21]. The measurements were conducted using the Quester Q10 system [22]. The QTF was connected to an impedance analyzer and a data-acquisition system. The impedance analyzer consists of analog-to-digital converters, amplifiers, a controller, and a signal generator. It can generate frequency sweeps at different amplitudes. Moreover, it can measure the real and imaginary components of impedance response at a rate of 0.5 million samples per second. Finally, the measured data are given in a comma-separated value format that may be analyzed in tools such as Matlab, Origin Lab, and Python. Furthermore, the instrument is capable of resonating a QTF at a specific frequency. Further details about the system are available in [22,23]. The Taguchi L9 design was used for statistical analysis, and the findings of this research showed that the exposure time and dose of the gamma source have a significant impact on the output response of resonance frequency. As a result, both time and dose were taken into account in this L9 Taguchi experiment. The details of input parameters of statistical analysis are shown in Supplementary File.

Three QTF types were used in the experiment: gold-coated QTF (QTF*g*), QTF coated with gold of larger surface area (QTF*gl*), and uncoated QTF (QTF). QTF*g* was coated on the surface with gold at a thickness of 100 nm [23] by sputtering deposition, whereas QTF*gl* was coated with gold at a tilted area of QTF (Figure 1).

The QTFs were irradiated under ambient humidity and temperature by Cs-137 (half-life: 2907 years, current activity: 0.78 μCi, dose rate: 7.64 μGy/h), which produces gamma rays with an energy of 662 keV at different time intervals (different doses). The gamma source was placed directly beside the QTF at a distance of 1.70 cm. The frequency was swept from 31 to 34.5 kHz for different exposure times (0, 1.0, and 2.5 h. Following each sensing measurements, the data were fit using the Lorentz function, and the resonance frequency was calculated using Equation (1) [20].

(1) $$\delta =\delta_{0}+\frac{2A}{\pi }\frac{\omega }{4{\left(\chi -\chi_{c}\right)}^{2}+\omega^{2}},$$

where δ is the real part of the impedance, $\delta_{0}$ is the offset, *A* is the area, ω is the difference between the values of the resonance frequency at full width at half maximum (FWHM), and $\chi_{c}$ is the resonance frequency at the maximum amplitude. The quality factor (*Q*) was calculated using the following equation [22]:

(2) $$Q=\frac{\chi_{c}}{\omega }.$$

A blank quartz substrate of 0.5 mm thick and 1 × 1 cm^2^ was used to investigate the optical properties of SiO_2_ before and after 3 h and 6 h radiation using another Cs-137 source of 3.42 µCi (dose rate 33.43 μGy/h). The optical properties were investigated by spectroscopic ellipsometry (SE, WVASE J. A. Woollam, Lincoln, NE, USA). The SE measurements were conducted in the range of 400–2500 nm with incidence angles of 55° (near Brewster angle of SiO_2_), 65°, and 75°.

## 3. Results and Discussion

After exposure to gamma radiation, a shift in the resonance frequencies of QTF was observed.

**Figure 2:**
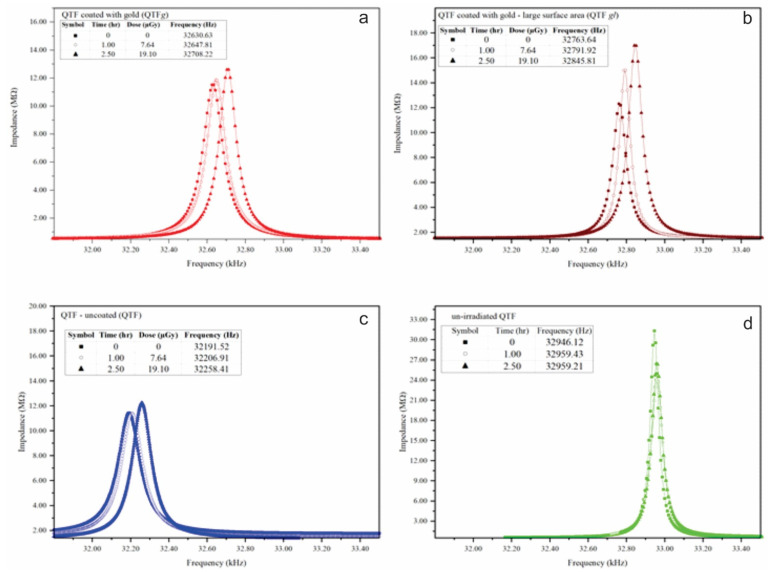
Resonance frequency shift of (**a**) QTF*g* QTF, (**b**) QTF*gl*, and (**c**) uncoated QTF in response to gamma radiation. (**d**) represents the QTF response without exposure to gamma radiation.

**Figure 3:**
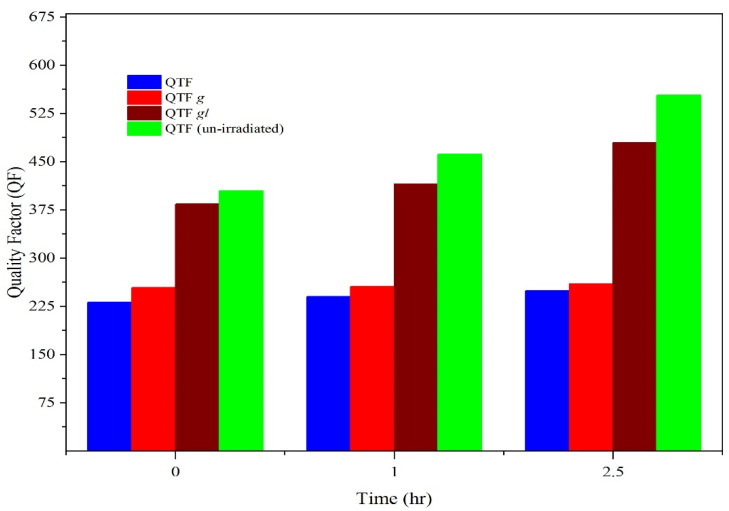
Change in the quality factor of QTFs with exposure time.

As shown in Figure 2a, there was a notable frequency shift in QTF*g* as the exposure time increased. The difference in the frequency shift (Δf) was 77.6 Hz. This change in resonance frequency was attributed to the increase in the quality factor (Figure 3). These results indicate that radiation energy can significantly affect the response of QTFs. To examine the effect of gold coating on the response of QTF, we conducted experiments on QTF*gl*. After 2.5 h, the resonance frequency increased from 32.76 to 32.84 kHz, approximately an 82.17 Hz frequency shift. The quality factor also increased from 378 to 490.

**Figure 4:**
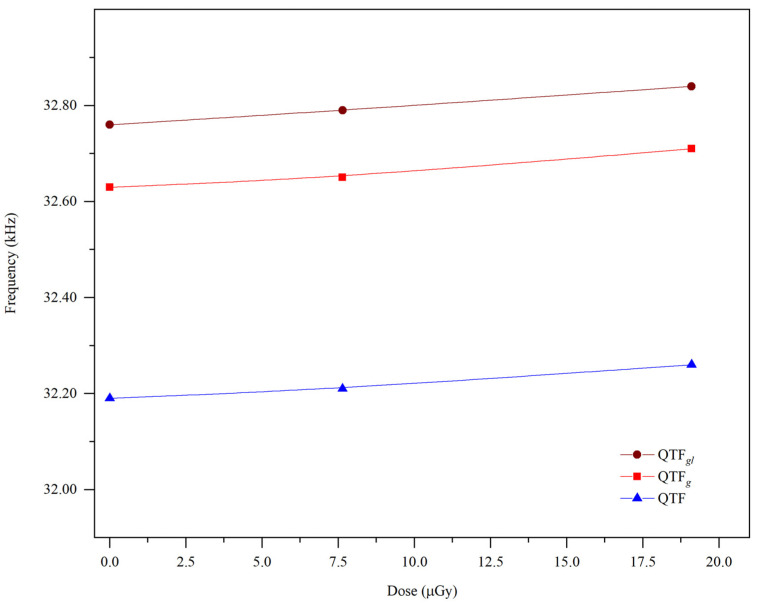
Comparison of resonance frequency shift of QTF*g*, QTF*gl*, and QTF as a function of different dose levels.

Although remarkable changes in resonance frequency and quality factor were observed in QTF, they were less than those observed on the face of QTF*g*. This result can be explained by comparing it with the frequency shift in QTF. As shown in Figure 2c, the frequency shift increased by about 66.8 Hz. Thus, the frequency shift is attributed to the interaction between gamma rays and quartz. In general, when exposed to high force or radiation energy, the fork coated with a higher amount of gold showed higher-frequency signals at its uppermost part [7]. For QTF*gl,* the shift in the resonance frequency was more than that of QTF*g*, indicating that gold acts as a shield to gamma rays, thus increasing the resonance frequency shift (as shown in Figure 4). Additionally, statistical analysis revealed that, when time and dosage levels increased, the frequency of the selected QTF systems (QTF*gl*, QTF*g*, and QTF) also increased. From the ANOVA tables (Supplementary File), the parameters with *p*-value < 0.05 were considered to be the significant and to actively contribute to the experimental responses. The model fit was found to be linear, and no interaction existed between the two input parameters. From this observation, it is evident that both input parameters efficiently contributed to its individual ability toward the active responses.

**Figure 5:**
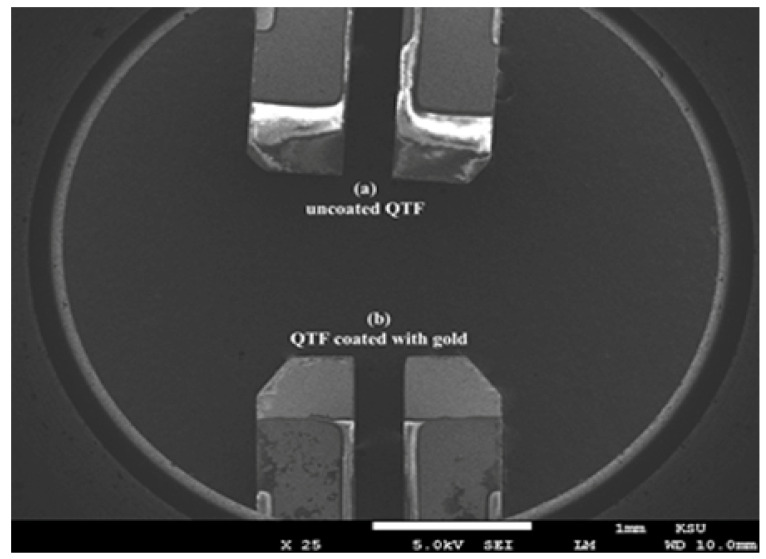
SEM images of (**a**) QTF and (**b**) QTF*g*.

**Figure 6:**
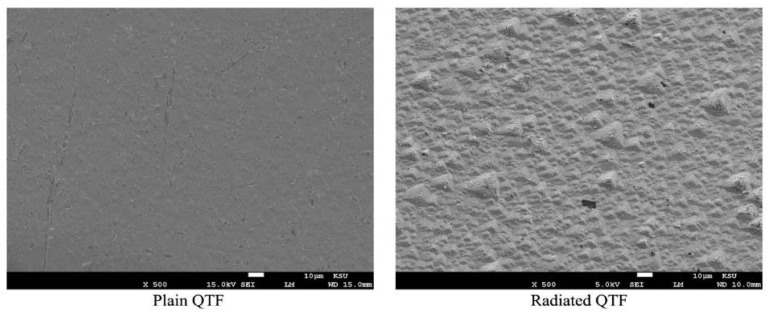
SEM images of QTF*g* before and after irradiation.

According to Insiripong et al., quartz changes color when exposed to gamma radiation [24]. Moreover, Sawakuchi and Okuno found from the characterization of thermoluminescence (TL) that quartz changes to a smoky color when irradiated by gamma rays, and this change is caused by color centers [25]. To verify this effect, QTF was observed under SEM after 2.5 h of radiation exposure. Figure 5 shows the SEM images of QTF and QTFg. Some changes could be observed on QTF compared to QTFg. The resonance frequency induced by the interaction between radiation and quartz could also be attributed to the presence of impurities or defects in the quartz [17]. These impurities in quartz increase its sensitivity to radiation, making it suitable as a radiation dosimeter [26]. To gain insight into the response of QTFg to the gamma radiation, irradiated and unirradiated QTF*g* were examined using SEM. Figure 6 shows that the gold thin film underwent a structural change, which consequently changed the resonance frequency of QTF. 

The effects of change in temperature and other environmental factors on the response of QTF were further investigated. Figure 2d shows a shift in the response frequency of QTF without exposure to gamma radiation. A frequency shift of only 12.7 Hz was observed. However, a change in quality factor was observed, which may be attributed to background radiations and thermal effects. The small change in frequency shift seen in Figure 2d confirms that the change in resonance frequency is mainly caused by the interaction between gamma radiation and QTF, indicating that the proposed QTF system has high stability over time and, thus, can be used for long-term measurements. The effect of radiation on other potential factors, such as circuit components (capacitors), was negligible, which is consistent with the result in [17]. 

At increased exposure times, the frequency of QTF was affected most by the radiation. To verify this, we exposed a SiO_2_ blank substrate to gamma rays and examined it by SE before and after each dose. This revealed the optical properties, such as the index of refraction as a function of wavelength and surface roughness (*S_rough_*) of QTF. 

**Figure 7:**
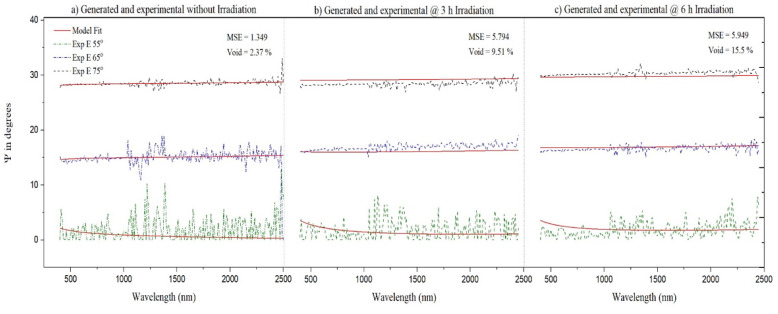
Spectroscopic ellipsometric Ψ for the SiO_2_ sample before and after 3 h and 6 h irradiation at incidence angles of 55°, 65°, and 75°.

The SE result was first fitted with that of a SiO_2_ model, but surface roughness was not in good agreement. Thus, the Cauchy model was applied for all samples, and all models were consistent, showing a decrease in the refractive index as the irradiation time increased. Hence, we incorporated voids within SiO_2_ since it could reduce the refractive index. Figure 7 shows the experimental and simulated *Ψ* before (Figure 7a) and after 3 h (Figure 7b) and 6 h (Figure 7c) irradiation at incidence angles of 55°, 65°, and 75° on SiO_2_ and in a wavelength range of 400–2500 nm. The accuracy of SE and the validity of our model were examined using low values of mean square error (MSE) and the agreement between the experimental and fitted data (Figure 7). 

The best fitting with the lowest MSE for each SE run was achieved when a mix of SiO_2_ was considered from the software library: thermal SiO_2_ optical constants and void with surface roughness [27]. The two materials were mixed by Bruggeman medium approximation (BEMA), enabling the fit of both SiO_2_ and void concentration [28]. The surface roughness was determined by assuming a layer of 50:50 of air (void) and an underneath layer. 

**Figure 8:**
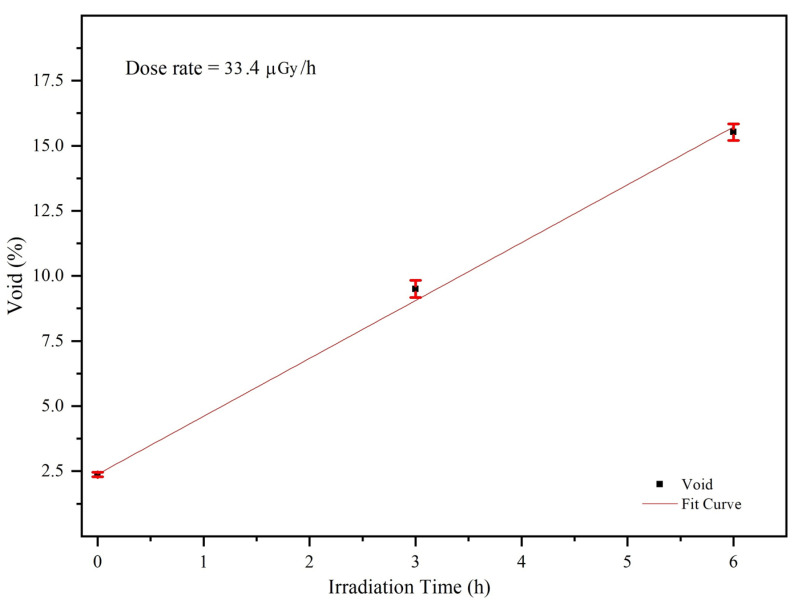
Void percentage in SiO_2_ as a function of irradiation time.

The fitted void percentage increased linearly with the irradiation dose (time) with *R^2^* = 0.997 (Figure 8). This is in good agreement with previous studies [29], showing the effect of gamma radiation on SiO_2_ defects. A change in the microstructure of a material affects its mechanical properties, resulting in a shift in the resonance frequency. This explains the change in the resonance frequency of QTF due to gamma irradiation. 

**Figure 9:**
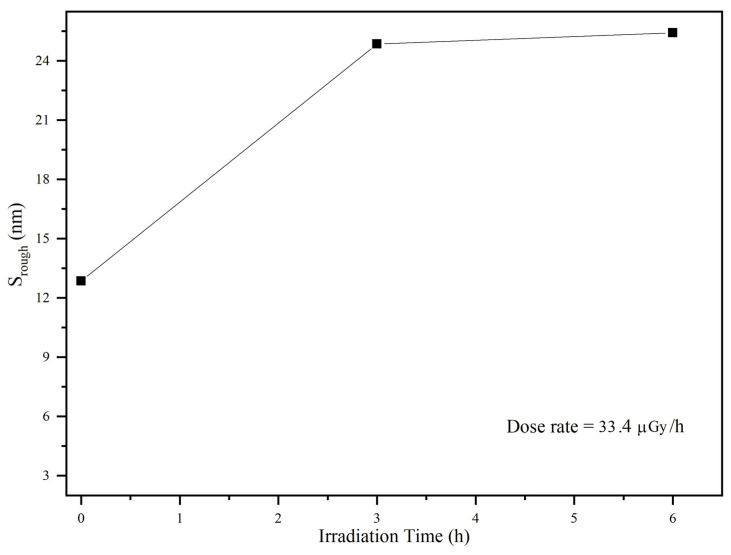
Optical surface roughness of SiO_2_ as a function of irradiation time.

Figure 9 shows the surface roughness *S_rough_* of the SiO_2_ surface at different irradiation doses (0, 3, and 6 h). The surface roughness increased as the irradiation time increased. This agrees well with the SEM images, showing protruded features on the QTF surface after irradiation. The increase in surface roughness was saturated, i.e., there was no significant change between the 3 h and 6 h irradiation. Considering both the void concentration and the surface roughness induced by gamma irradiation, we infer that the gamma ray-induced voids in SiO_2_ significantly affect the frequency shift in QTF, whereas the gamma ray-induced surface roughness has a much lower impact on the frequency shift.

According to these results, QTFs exhibit promising behavior when exposed to gamma radiation. Therefore, they have great potential in gamma detection. They also show the possibility of extending the application of QTFs to other radiation (alpha and beta particles).

## 4. Conclusions

In this study, we investigated the potential of QTFs in sensing gamma radiation. Different QTFs were studied to deeply understand the behavior and factors affecting the QTF response. The results demonstrate that QTF can detect gamma radiation by changing the resonance frequency. The magnitude of the change in resonance frequency varied among the QTFs; the largest shift was recorded in QTF*gl* and QTF*g* as compared to uncoated QTF. This result indicates that the thickness of gold played a vital role in increasing the sensitivity of the QTF to gamma rays. SE measurements of SiO_2_ revealed a linear change in the void percentage in SiO_2_ with the irradiation time, indicating that the frequency shifts in QTF are mainly attributed to the gamma–SiO_2_ interaction. These results show that QTF is promising in dosimetry applications as a simple, inexpensive, and rapid method for detecting radiation. Overall, as compared to other existing techniques, this proposed method represents a good alternative technique, which can provide ultrasensitive, rapid, cost-efficient, and small-size detection of nuclear radiation. It also opens avenues for exploring other types of radiation, such as alpha and beta particles. In order for QTFs to be used in practical application, further studies that can address more aspects of the response of the QTF should be conducted. Such studies include using different thicknesses of the coating layer and changing the gamma source to other sources of different energy. 

## Data Availability

Not applicable.

## Supplementary Materials

The following are available online at https://www.mdpi.com/article/10.3390/ma14227035/s1, Figure S1: Residual Plots for QTF: resonance frequency vs time (hr), Dose (µGy) level of gamma source*,* Figure S2: Residual Plots for QTFgl: resonance frequency vs time (hr), Dose (µGy) level of gamma source, Figure S3: Residual Plots for QTFgl: resonance frequency vs time (hr), Dose (µGy) level of gamma source, Figure S4: Response optimizer for time, dose level vs resonance frequency, Table S1: Design of Experiment with Main Variables, Table S2: Taguchi Experimental Design Test Matrix (L9) for Detection of gamma radiation as a function of dose and time duration, Table S3: ANOVA for resonance frequency response of QTFg vs time (hr), dose (µGy) level, Table S4: ANOVA for resonance frequency response of QTFgl vs time (hr), dose (µGy) level, Table S5: ANOVA for resonance frequency response of QTF vs time (hr), dose (µGy) level.
