# Supplementary material for: Quartz Tuning Fork Sensor-Based Dosimetry for Sensitive Detection of Gamma Radiation
Source: Materials (Basel). 2021 Nov 19;14(22):7035. doi: 10.3390/ma14227035 (PMC8619227; doi:10.3390/ma14227035)
Supplement: Supplementary file 1 [file materials-14-07035-s001.zip › materials-1367575-supplementary.pdf]

materials

MDPI

Supplementary File

Quartz Tuning Fork Sensor-Based Dosimetry for Sensitive Detection of Gamma Radiation

Nadyah Alanazi [1,†], Abdullah N. Alodhayb [1,2,3,†], Atheer Almutairi [1], Hanan Alshehri [1], Sarah AlYemni [1], Ghadah Alsowygh [1], Sabaa Abdulmawla [1], Khaled Shamma [1,3], Hamad Albrithen [1,2,3,4], Muthumareeswaran Muthuramamoorthy [3] and Aljawhara H. Almuqrin [5,*]

[1] Department of Physics and Astronomy, College of Science, King Saud University, Riyadh 11451, Saudi Arabia; nalenazi@ksu.edu.sa (N.A.); aalodhayb@ksu.edu.sa (A.N.A.); 438202676@student.ksu.edu.sa (A.A.); hoalshehri@KSU.EDU.SA (H.A.); salyemni@KSU.EDU.SA (S.A.); galsowygh@ksu.edu.sa (G.A.); stabdulmawla@ksu.edu.sa (S.A.); khaledshamma@hotmail.com (K.S.); brithen@ksu.edu.sa (H.A.)

[2] Research Chair for Tribology, Surface, and Interface Sciences, Department of Physics and Astronomy, College of Science, King Saud University, Riyadh 11451, Saudi Arabia

[3] King Abdullah Institute for Nanotechnology, King Saud University, Riyadh 11451, Saudi Arabia; mramamoorthy@ksu.edu.sa

[4] K.A.CARE Energy Research and Innovation Center at Riyadh, Riyadh 11451, Saudi Arabia

[5] Department of Physics, College of Science, Princess Nourah bint Abdulrahman University, Riyadh, Saudi Arabia

* Correspondence: ahalmoqren@pnu.edu.sa

† Authors equally contributed to this work.

Citation: Alanazi, N.; Alodhayb, A.; Almutairi, A.; Alshehri, H.; AlYemni, S.; Alsowygh, G.; Abdulmawla, S.; Shamma, K.; Albrithen, H.; Muthuramamoorthy, M.; et al. Quartz Tuning Fork Sensor-Based Dosimetry for Sensitive Detection of Low-Dose Gamma Radiation. *Materials* **2021**, *14*, 7035. https://doi.org/10.3390/ma14227035

Academic Editor: Qiang Xu, Salvatore Tudisco, Francesco Caridi, Valery V. Tuchin

Received: 19 August 2021
Accepted: 15 November 2021
Published: 19 November 2021

Publisher's Note: MDPI stays neutral with regard to jurisdictional claims in published maps and institutional affiliations.

Copyright: © 2021 by the authors. Licensee MDPI, Basel, Switzerland. This article is an open access article distributed under the terms and conditions of the Creative Commons Attribution (CC BY) license (http://creativecommons.org/licenses/by/4.0/).

Statistical Analysis

The number of experimental runs required to run the experiment was designed using the Taguchi L9 design. From the literature, among the dominant parameters of detecting radiation sources from gamma, time and dose played an vital role in contributing the output response resonance frequency. So, time and dose have been considered in this L9 Taguchi experiment.

Table S1. Design of Experiment with Main Variables.

Input Parameters	Levels	Actual Values
Time	(−1, 0, +1)	(0, 1, 2.5)
Dose	(−1, 0, +1)	(0, 7.64, 19.10)

Experimental Data Sets

Table S2. Taguchi Experimental Design Test Matrix (L9) for Detection of gamma radiation as a function of dose and time duration.

Time (hr)	Dose (μGy)	QTF*g*	QTF*gl*	QTF
0.0	0.00	32.62	32.73	32.16
0.0	7.64	32.63	32.76	32.18
0.0	19.10	32.64	32.77	32.19
1.0	0.00	32.65	32.78	32.20
1.0	7.64	32.67	32.79	32.21
1.0	19.10	32.69	32.81	32.22
2.5	0.00	32.71	32.82	32.24
2.5	7.64	32.73	32.84	32.25
2.5	19.10	32.75	32.86	32.26

Results

The regression equation (1) of

QTF*g* = 32.6767 - 0.04667 Time (hr)_0.0 - 0.00667 Time (hr)_1.0 + 0.05333 Time (hr)_2.5
- 0.01667 Dose (μGy) _ 0.00 + 0.00000 Dose (μGy)_7.64 + 0.01667 Dose (μGy)_19.10

Table S3. ANOVA for resonance frequency response of QTFg vs time (hr), dose (μGy) level.

Source	*df*	Sum of Squares	Mean of Squares	F- Value	P-Value	
Time (hr)	2	0.015200	0.007600	228.00	0.000	Significant
Dose (μGy)	2	0.001667	0.000833	25.00	0.005	Significant

The regression equation (2) of

QTF*gl* = 32.7956 - 0.04222 Time (hr)_0.0 - 0.00222 Time (hr)_1.0 + 0.04444 Time (hr)_2.5
- 0.01889 Dose (μGy)_0.00 + 0.00111 Dose (μGy)_7.64 + 0.01778 Dose (μGy)_19.10

Table S4. ANOVA for resonance frequency response of QTFgl vs time (hr), dose (μGy) level.

Source	*df*	Sum of Squares	Mean of Squares	F- Value	P-Value	
Time (hr)	2	0.011289	0.005644	203.20	0.000	Significant
Dose (μGy)	2	0.002022	0.001011	36.40	0.003	Significant

The regression equation (3) of

QTF = 32.2122 - 0.03556 Time (hr)_0.0 - 0.00222 Time (hr)_1.0 + 0.03778 Time (hr)_2.5
- 0.01222 Dose (μGy)_0.00 + 0.00111 Dose (μGy)_7.64 + 0.01111 Dose (μGy)_19.10

Table S5. ANOVA for resonance frequency response of QTF vs time (hr), dose (μGy) level.

Source	*df*	Sum of Squares	Mean of Squares	F- Value	P-Value	
Time hr)	2	0.008089	0.004044	364.00	0.000	Significant
Dose (μGy)	2	0.000822	0.000411	37.00	0.003	Significant

From the ANOVA tables, the results inferred that the parameters with p value <0.05 is considered to be the significant and actively contributed to the experimental responses. The model fit found to be linear and there is no interaction exists between the two input parameters. From this observation, it is evident that both input parameters efficiently contributed with its individual ability towards the active responses under desirability 1. In addition, the predicted all analysis R^2 value (99.42 ± 0.3) is closely matches with adjusted R^2 (96.72 ± 0.1) which makes the data scatters of actual and predicted as very close fit data.

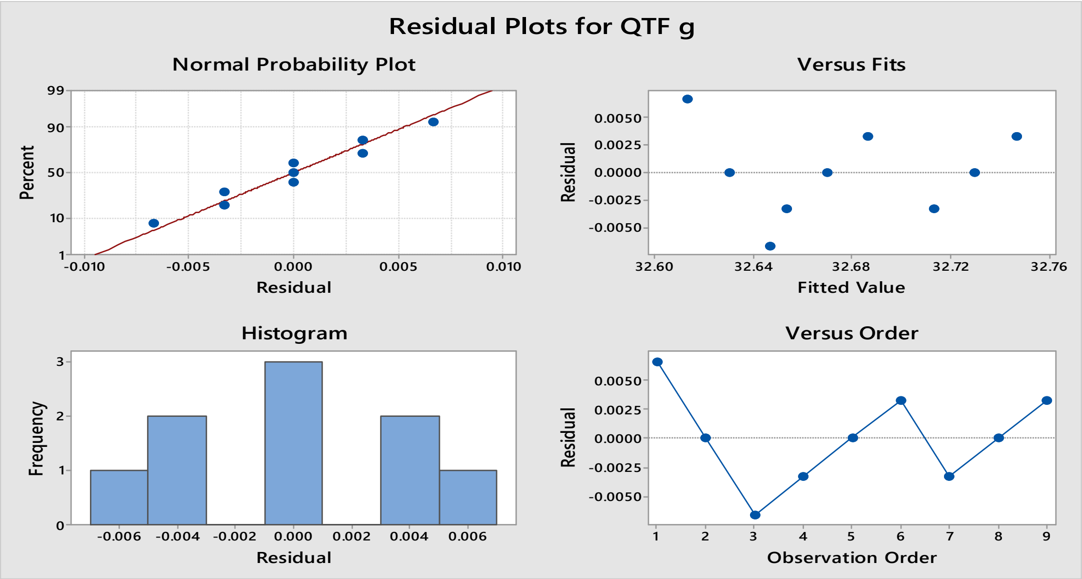

Figure S1. Residual Plots for QTF : resonance frequency vs time (hr), Dose (**μ**Gy) level of gamma source.

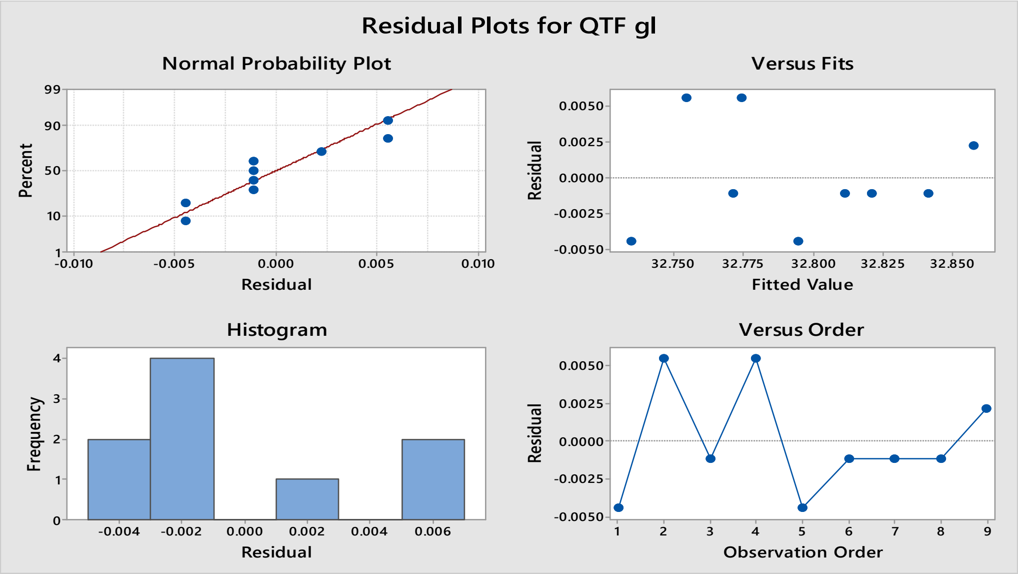

Figure S2. Residual Plots for QTFgl : resonance frequency vs time (hr), Dose (**μ**Gy) level of gamma source.

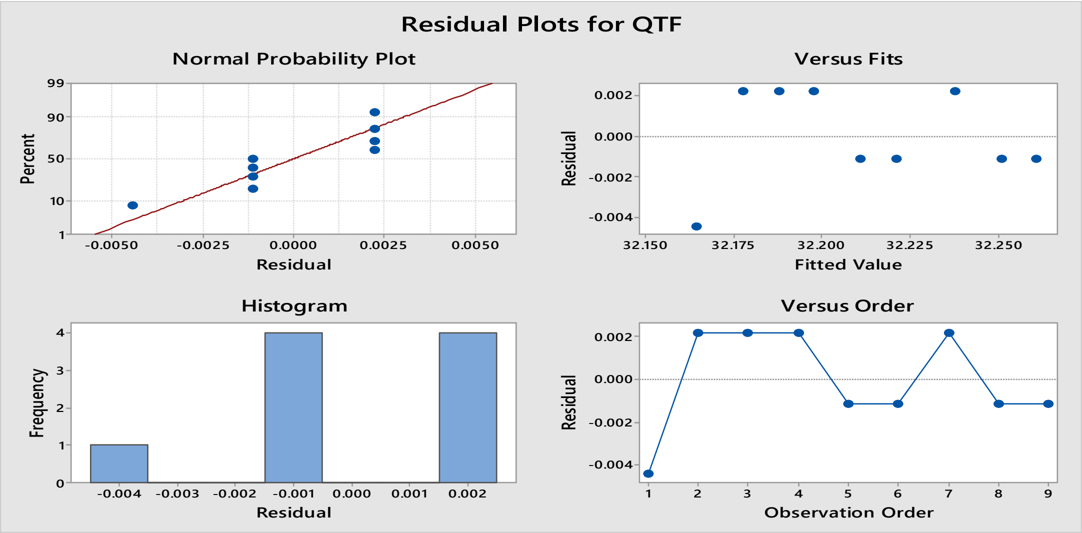

Figure S3. Residual Plots for QTFgl : resonance frequency vs time (hr), Dose (µGy) level of gamma source.

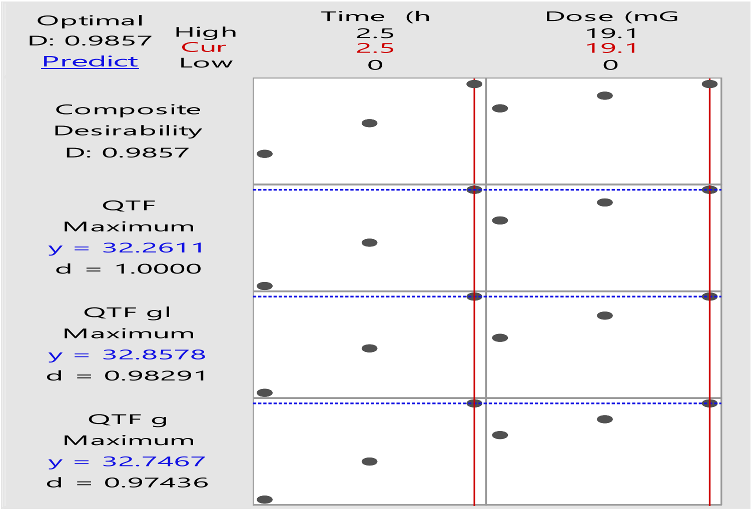

Figure S4. Response optimizer for time, dose level vs resonance frequency.

From the response optimizer, it was concluded that increase in the time and dose obviously increasing the frequency of the selected QTF systems (QTF*gl*, QTF*g* and QTF).
